# Toll-Like Receptor 5 of Golden Pompano *Trachinotus ovatus* (Linnaeus 1758): Characterization, Promoter Activity and Functional Analysis

**DOI:** 10.3390/ijms21165916

**Published:** 2020-08-18

**Authors:** Ke-Cheng Zhu, Meng Wu, Dian-Chang Zhang, Hua-Yang Guo, Nan Zhang, Liang Guo, Bao-Suo Liu, Shi-Gui Jiang

**Affiliations:** 1 Key Laboratory of South China Sea Fishery Resources Exploitation and Utilization, Ministry of Agriculture and Rural Affairs, South China Sea Fisheries Research Institute, Chinese Academy of Fishery Sciences, Guangzhou 510300, China; zkc537@163.com (K.-C.Z.); 13871270376@163.com (M.W.); guohuayang198768@163.com (H.-Y.G.); 398730316@163.com (N.Z.); zsdxgl@163.com (L.G.); liubaosuo343@163.com (B.-S.L.); jiangsg@21cn.com (S.-G.J.); 2 Guangdong Provincial Engineer Technology Research Center of Marine Biological Seed Industry, Guangzhou 510300, China; 3 Tropical Aquaculture Research and Development Center, South China Sea Fisheries Research Institute, Chinese Academy of Fishery Sciences, Sanya 572018, China; 4 Sanya Tropical Fisheries Research Institute, Sanya 510300, China

**Keywords:** *Trachinotus ovatus*, promoter activity, Toll-like receptor 5, binding assay

## Abstract

Toll-like receptors (TLRs), as important pattern recognition receptors, represent a significant component of fish immune systems and play an important role in resisting the invasion of pathogenic microorganisms. The TLR5 subfamily contains two types of TLR5, the membrane form of TLR5 (TLR5M) and the soluble form of TLR5 (TLR5S), whose detailed functions have not been completely elucidated. In the present study, we first identified two genes, *TLR5M* (*ToTLR5M*) and *TLR5S* (*ToTLR5S*), from golden pompano (*Trachinotus ovatus*). The full-length *ToTLR5M* and *ToTLR5S* cDNA are 3644 bp and 2329 bp, respectively, comprising an open reading frame (ORF) of 2673 bp, encoding 890 amino acids, and an ORF of 1935 bp, encoding 644 amino acids. Both the *ToTLR5s* possess representative TLR domains; however, only *ToTLR5M* has transmembrane and intracellular TIR domains. Moreover, the transcription of two *ToTLR5s* was significantly upregulated after stimulation by polyinosinic:polycytidylic acid (poly (I:C)), lipopolysaccharide (LPS), and flagellin in both immune-related tissues (liver, intestine, blood, kidney, and skin) and nonimmune-related tissue (muscle). Furthermore, the results of bioinformatic and promoter analysis show that the transcription factors GATA-1 (GATA Binding Protein 1), C/EBPalpha (CCAAT Enhancer Binding Protein Alpha), and ICSBP (Interferon (IFN) consensus sequence binding protein) may play a positive role in moderating the expression of two *ToTLR5s*. Overexpression of *ToTLR5M* and *ToTLR5S* notably increases *NF-κB* (nuclear factor kappa-B) activity. Additionally, the binding assay revealed that two rToTLR5s can bind specifically to bacteria and pathogen-associated molecular patterns (PAMPs) containing *Vibrio harveyi*, *Vibrio anguillarum*, *Vibrio vulnificus*, *Escherichia coli*, *Photobacterium damselae*, *Staphylococcus aureus*, *Aeromonas hydrophila*, LPS, poly(I:C), flagellin, and peptidoglycan (PGN). In conclusion, the present study may help to elucidate the function of ToTLR5M/S and clarify their possible roles in the fish immune response to bacterial infection.

## 1. Introduction

Toll-like receptors (TLRs) play an important role in host defense in both invertebrates and vertebrates [1]. The TLR family, a key pathogen recognition receptor (PRR), primarily participates in the innate immune and acquired immune systems. In higher vertebrates, the TLR family is regarded as a pivotal mediator that activates innate immunity and develops antigen-specific acquired immunity against invading microorganisms and pathogens, such as peptidoglycan, lipoprotein, lipopolysaccharide (LPS), flagellin, and polyinosinic:polycytidylic acid (poly(I:C)) [2,3,4]. To date, as TLRs are type I transmembrane proteins, 13 types of TLRs have been reported in mammals [5,6]. According to cellular localization, TLRs are classified into two major subfamilies: the cell surface subfamily (TLRs 1, 2, 4, 5, 6, and 10) and the endolysosomal compartment subfamily (TLRs 3, 7, 8, and 9) [4]. TLRs include three typical domains: the extracellular, transmembrane, and intracellular domains [7]. The extracellular domain is composed of several N-terminal leucine-rich repeats (LRRs) and is involved in recognition of pathogen-associated molecular patterns (PAMPs) [8]. Moreover, the intracellular domain possesses a representative Toll/IL-1 (Interleukin-1) receptor (TIR) domain that is analogous to the IL1R family intracellular domain [9] and plays a pivotal role in signal transduction.

As a TLR family member, TLR5 is a key PRR and can recognize PAMPs, such as the bacterial flagellum protein, by touching their flagellin [10,11,12,13]. TLR5 is reasonable for flagellin-mediated *NF-κB* activation by the MyD88-dependent pathway in the cellular membrane [14]. Two types of TLR5 are found in teleosts: the membrane form of TLR5 (TLR5M) and the soluble form of TLR5 (TLR5S) [15,16,17,18]. In other fish species, only TLR5M in *Cirrhinus mrigala*, *Danio rerio*, *Ctenopharyngodon idelus*, *Oplegnathus fasciatus*, *Pangasianodon hypophthalmus*, and *Paralichthys olivaceus* [13,19,20,21,22,23] or TLR5S in *Onchorhynchus mykiss*, *Salmo salar*, *Ictalurus punctatus*, *Cynoglossus semilaevis*, and *Miichthys miiuy* [16,24,25,26,27] have been isolated and reported. Furthermore, TLR5M is composed of the TIR domain transmembrane region and the LRR domain, but TLR5S has no TIR domain or transmembrane region by comparison. A recent study showed that TLR5M identified flagellin, and NF-кB was activated to induce some immune response genes and TLR5S [16]. TLR5S was induced to recognize flagellin in the fluid phase and later bound to TLR5M to amplify the signal cascade [17].

The golden pompano *Trachinotus ovatus* (Linnaeus 1758), Carangidae, Perciformes, is widely distributed in the Asia–Pacific region. This fish is popular because of its fast growth and high-quality flesh and is known as an important commercial fish in China [28,29]. However, in *T. ovatus*, a high death rate is attributed to viral and bacterial infections [30]. To characterize the host immune defense and host–pathogen relationships of *TLR5* in *T. ovatus* (*ToTLR5*), we determined the role of *ToTLR5* after stimulation with poly(I:C), LPS, and flagellin, and the genomic sequence, expression pattern, and transcriptional regulation of *ToTLR5* were also determined. Our results suggest that ToTLR5 may play an important role as a PRR in the immune response to pathogen infections and may be involved in the NF-кB activation pathway.

## 2. Results

### 2.1. Sequence Characterization of ToTLR5M and ToTLR5S

**Figure 1:**
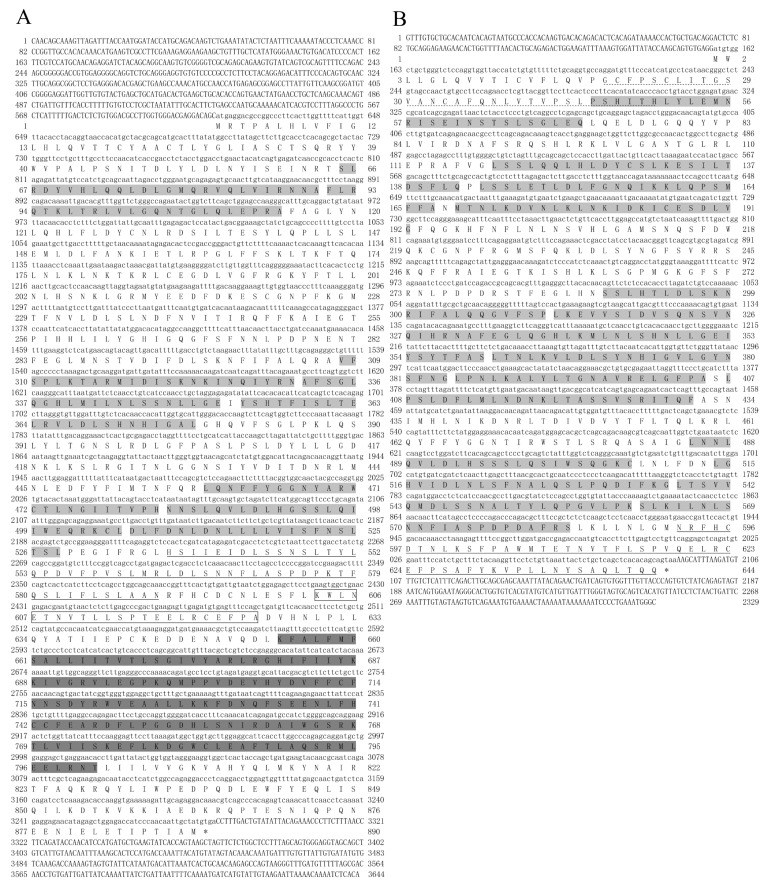
The full-length cDNA and deduced amino acid sequences of ToTLR5M (**A**) and ToTLR5S (**B**). The leucine-rich repeat (LRR) and LRR-NT domains are highlighted in light gray and by dotted lines, respectively. The LRR-CT domains are underlined in ToTLR5S (**B**). The transmembrane region is indicated by box and the Toll/IL-1 receptor (TIR) domain is marked with gray; termination codon is indicated with “*”.

**Figure 2:**
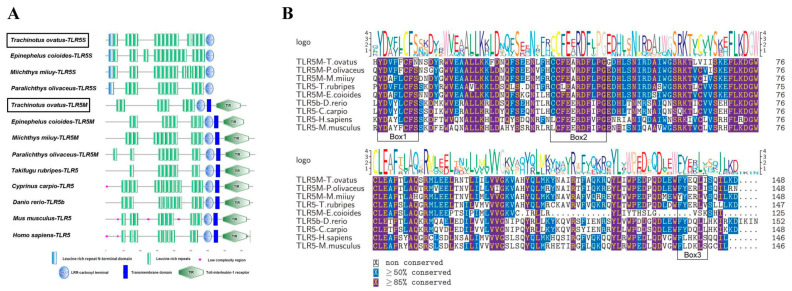
The domain features of the membrane form of TLR5 (TLR5M) and the soluble form of TLR5 (TLR5S) among vertebrates. (**A**) Multiple alignment of TLR5 deduced amino acid sequences. LRR represents leucine-rich repeats, red represents low-complexity region, LRR-CT represents LRR C-terminal region, blue represents transmembrane region, TIR represents Toll/interleukin-I receptor domain. (**B**) The amino acid sequence alignment of TLR5 TIR domains in various species. The GenBank accession numbers are shown in Table S1.

The cDNA sequence of *ToTLR5M* is 3644 bp, including 612 bp of the 5′ untranslated region (5′-UTR); a 2673 bp open reading frame (ORF) encoding a polypeptide of 890 amino acids (GenBank accession number: MT596697; Figure 1A); and a 359 bp 3′-UTR, with a predicted molecular weight of 96.31 kDa and a theoretical isoelectric point of 5.94. The cDNA sequence of *ToTLR5S* is 2329 bp, including 156 bp of the 5′ untranslated region (5′-UTR); a 1935 bp ORF encoding a polypeptide of 644 amino acids (GenBank accession number: MT596698; Figure 1B); and a 238 bp 3′-UTR, with a predicted molecular weight of 72.21 kDa and a theoretical isoelectric point of 8.84. Moreover, the amino acid sequence of *ToTLR5M* contains typical TLR protein domains, eight LRR domains (65–528 aa), one LRR-CT domain (537–590 aa), one transmembrane domain (603–625 aa), and one intracellular TIR domain (654–801 aa) (Figure 1A and Figure 2A). The amino acid sequence of *ToTLR5S* includes 1 LRR-NT domain (19–50 aa), 14 LRR domains (45–583 aa), and 1 LRR-CT domain (592–644 aa) (Figure 1B and Figure 2A). Furthermore, in TLR5M, the result of amino acid sequence alignment of the TIR domain showed that this region contains three conserved functional boxes (Box1, Box2, and Box3) (Figure 2B). Additionally, comparison of the exon–intron organization of TLR5 shows that the genomic sequences of *ToTLR5M* and *ToTLR5S* include four exons, three introns, two exons, and one intron, respectively (Figure S1).

### 2.2. Tissue Expression of ToTLR5M and ToTLR5S

**Figure 3:**
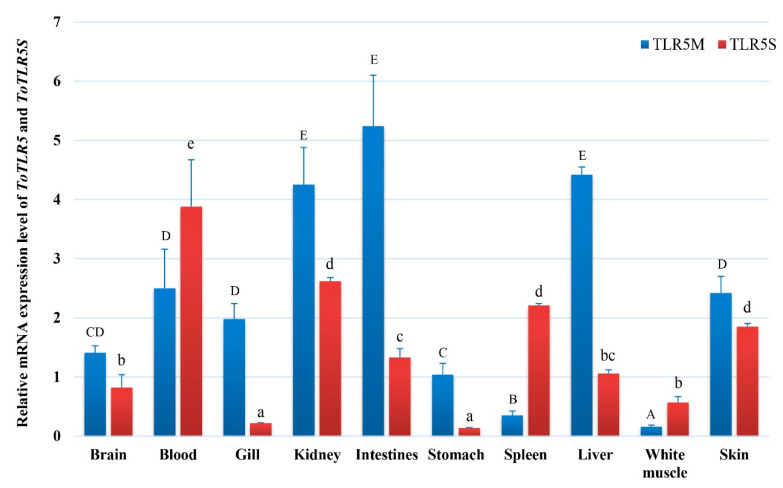
The tissue expression of the *ToTLR5M* and *ToTLR5S* genes. The tissues included kidney, liver, stomach, spleen, intestine, brain, skin, gill, muscle, and blood. Elongation factor 1 alpha (*EF-1α*) acted as an internal reference to calibrate the cDNA templates. Mean ± standard error (SE) (*n* = 3) of each mRNA quantity was shown for each tissue examined. Different uppercase or lowercase letters indicate significant differences (*p* < 0.05).

To confirm the role of *ToTLR5M* and *ToTLR5S* in healthy fish, we used qRT-PCR to detect the mRNA expression levels in 10 tissues (Figure 3). Two *ToTLRs* were constitutively expressed in all tissues analyzed, with varied expression levels being observed. *ToTLR5M* is highly expressed in the intestine, kidney, and liver, followed by the blood, skin, gill, brain, and stomach, with lower expression levels being observed in the spleen and white muscle (*p* < 0.05). However, *ToTLR5S* is highly expressed in the blood, kidney, spleen, and skin, followed by the intestine, liver, brain, and white muscle, with lower mRNA levels being observed in the gill and stomach (*p* < 0.05).

**Figure 4:**
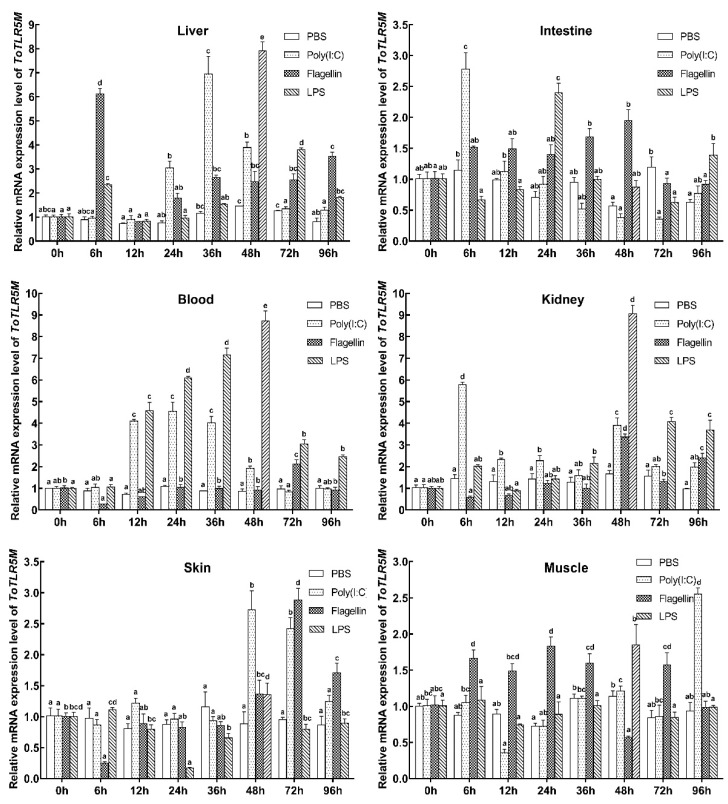
*ToTLR5M* expressions in different tissues (liver, kidney, intestine, skin, muscle, and blood) after phosphate-buffered saline (PBS), flagellin, poly(I:C), and LPS challenge. *EF-1α* acted as an internal control to calibrate the cDNA templates. All data are expressed as mean ± SE. Different letters show significant differences (*p* < 0.05).

**Figure 5:**
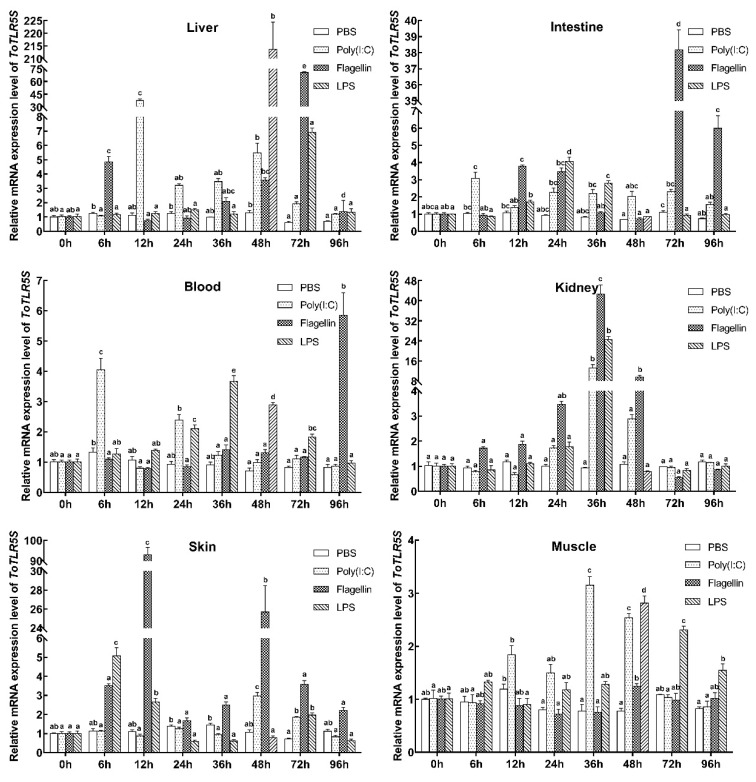
*ToTLR5S* expressions in different tissues (liver, kidney, intestine, skin, muscle, and blood) after PBS, flagellin, poly(I:C), and LPS challenge. *EF-1α* acted as an internal control to calibrate the cDNA templates. All data are expressed as mean ± SE. Different letters show significant differences (*p* < 0.05).

To further investigate the role of *ToTLR5M* (Figure 4) and *ToTLR5S* (Figure 5) in the immune response, we also used qRT-PCR to investigate gene expression in response to poly(I:C), LPS, and flagellin challenges. In comparison to the control group, the mRNA levels of *ToTLR5M* were markedly increased in response to poly(I:C), LPS, and flagellin challenges in the immune- and nonimmune-related tissues, suggesting the possible role of *ToTLR5M* in defense against pathogenic microbes. As shown in Figure 4, *ToTLR5M* transcription was more sensitive in the liver, blood, and kidney than in the intestine, skin, and muscle, suggesting a dramatic increase of 7.92-fold, 8.73-fold, and 9.07-fold in the liver, blood, and kidney after infection with LPS, respectively, compared to the control. Nevertheless, there was a dramatic increase of 2.78-fold, 2.73-fold, and 2.55-fold in the intestine, skin, and muscle after infection with poly(I:C), flagellin, and poly(I:C), respectively, compared to the control.

In comparison to the control group, *ToTLR5S* expression was upregulated by poly(I:C), LPS, and flagellin challenges in the immune- and nonimmune-related tissues (Figure 5). Notably, *ToTLR5S* was more responsive in the liver, skin, intestine, and kidney than in the other two tissues, showing remarkable increases of 213.64-fold, 93.08-fold, 38.16-fold, and 42.65-fold in the liver, skin, intestine, and kidney after challenge with LPS, flagellin, flagellin, and flagellin, respectively, compared to the control. Nevertheless, compared to the control, there was a dramatic increase of 5.85-fold and 3.15-fold in the blood and muscle after infection with flagellin and poly(I:C), respectively.

### 2.3. Promoter Activity of Two ToTLR5s

**Figure 6:**
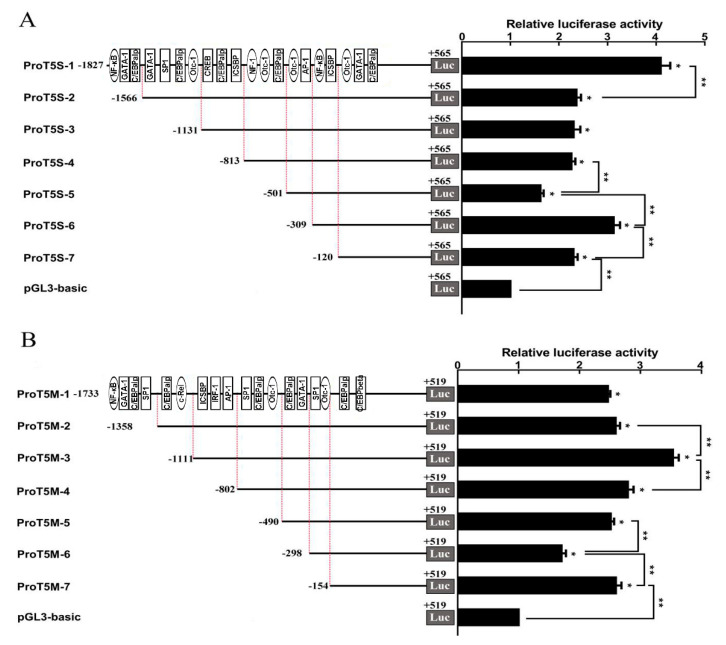
Promoter activity analysis of *ToTLR5M* (**A**) and *ToTLR5S* (**B**). (**A**) Seven recombinant plasmids, denoted as ProT5M-1 (−1827 to +565), ProT5M-2 (−1566 to +565), ProT5M-3 (−1131 to +565), ProT5M-4 (−813 to +565), ProT5M-5 (−501 to +565), ProT5M-6 (−309 to +565), and ProT5M-7 (−120 to +565) were constructed and transfected into *Trachinotus ovatus* snout tissue (GPS) cells. (**B**) Seven recombinant plasmids, denoted ProT5S-1 (−1733 to +519), ProT5S-2 (−1358 to +519), ProT5S-3 (−1111 to +519), ProT5S-4 (−802 to +519), ProT5S-5 (−490 to +519), ProT5S-6 (−298 to +519), and ProT5S-7 (−154 to +519) were constructed and transfected into GPS cells. Different color boxes indicate binding sites located in different truncation regions. All data are expressed as mean ± SE in the picture (*n* = 5). * indicates significant differences (*p* < 0.05). ** indicates extremely significant differences (*p* < 0.01).

The 5′-flanking fragment of two *ToTLR5* genes lacked both the TATA box and CAAT box. The transcription factor binding sites for C/EBPalpha, C/EBPbeta, Sp1, AP-1, NF-1, Oct-1, GATA-1, NF-κB, ICSBP, c-Rel, and IRF-1 could be predicted in the 5’-flanking sequence of the *ToTLR5M* gene (Figure S2A). Deletion of the sequences from −1827 to −1566 bp, −813 to −501 bp, −309 to −120 bp, and −120 to +1 bp of *ToTLR5M* significantly decreased the relative luciferase activity, indicating that transcription factors of NF-κB, GATA-1, C/EBPalpha, NF-1, Oct-1, and ICSBP played a positive role in the regulatory effect. Furthermore, there was no significant difference after the deletion of the sequences from −1566 to −813 bp. However, the relative luciferase activity was increased by deleting the sequences of −501 to −309 bp (Figure 6A).

The transcription factor binding sites for C/EBPalpha, C/EBPbeta, C/EBPgamma, Sp1, AP-1, Oct-1, GATA-1, IRF1, NF-κB, ICSBP, NF-1, Sox-2, SGF-3, CREB, and NF-κ could be predicted in the 5’-flanking sequence of the *ToTLR5M* gene (Figure S2B). Deletion of the sequences from −1111 to −802 bp, −490 to −298 bp, and −154 to +1 bp of *ToTLR5S* significantly decreased the relative luciferase activity, indicating that the transcription factors ICSBP, IRF1, AP-1, GATA-1, C/EBPalpha, and C/EBPbeta played positive roles in the transcriptional regulatory system. Moreover, there was no significant difference after the deletion of the sequences from −1733 to −1358 bp and −802 to −490 bp. However, the relative luciferase activity was increased by deleting the sequences of −1358 to −1111 bp and −298 to −154 bp (Figure 6B).

### 2.4. Effect of Overexpression of Two ToTLR5s on NF-κB Activity

**Figure 7:**
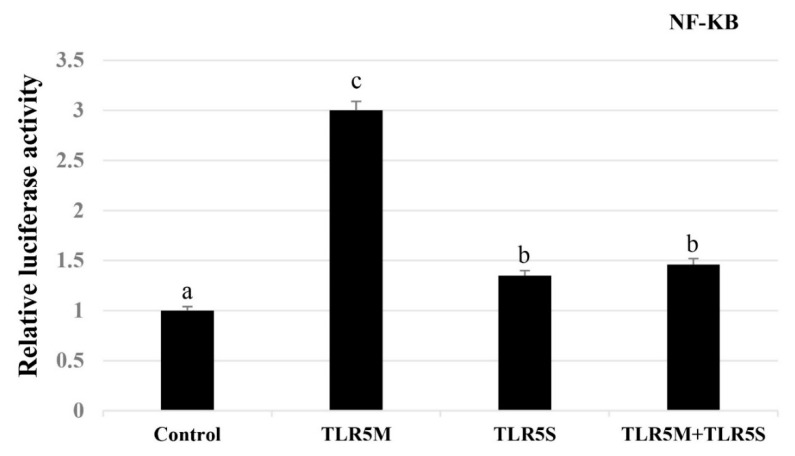
Overexpression of two *ToTLR5* genes altered the expression levels of NF-κB. The cells were transfected with an empty vector or *ToTLR5s*-pcDNA3.1. Each of them was co-transfected with an NF-κB reporter plasmid. All data are expressed as mean ± SE. Different letters show significant differences (*p* < 0.05).

To further confirm the interaction of the two *ToTLR5s* with *NF-κB*, we determined the influence of *ToTLR5M* or *ToTLR5S* overexpression on *NF-κB* transcription. We performed a luciferase reporter assay using golden pompano *Trachinotus ovatus* snout tissue (GPS) cells transiently cotransfected with NF-κB reporter vector and ToTLR5M-pcDNA3.1 or ToTLR5S-pcDNA3.1 individually or both *ToTLR5M* and *ToTLR5S* together. The results showed that overexpression of *ToTLR5M* or *ToTLR5S* can significantly enhance NF-кB activity (*p* < 0.01), and overexpression of both ToTLR5M and ToTLR5S can also significantly enhance NF-кB activity (*p* < 0.01) (Figure 7). The relative luciferase activity of *NF-κB* was highest with cotransfection of ToTLR5M.

### 2.5. Binding of Two rToTLR5s to Bacteria and PAMPs

**Figure 8:**
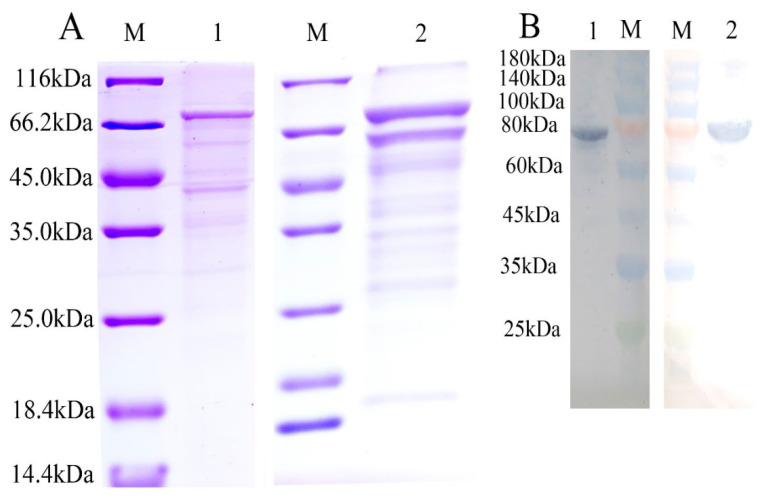
SDS-PAGE (**A**) and Western blot (**B**) analysis of two purified recombinant ToTLR5s. (**A**) Lane M: standard protein marker; lane A1: purified recombinant ToTLR5M; lane A2: purified recombinant ToTLR5S. (**B**) Lane M: standard protein marker; lane B1: rToTLR5M; lane B2: rToTLR5S.

The two rToTLR5 proteins were expressed in the pET-sumo vector in *Escherichia coli* (Rosetta DE3). SDS-PAGE (Figure 8A) and Western blotting analysis (Figure 8B) showed that recombinant ToTLR5M and ToTLR5S proteins were successfully expressed (Figure 8). The recombinant proteins were purified using Ni-NTA resin. The observed molecular weights of rToTLR5M (≈76 kDa) and rToTLR5S (≈85 kDa) were close to the predicted molecular weights of the proteins.

**Figure 9:**
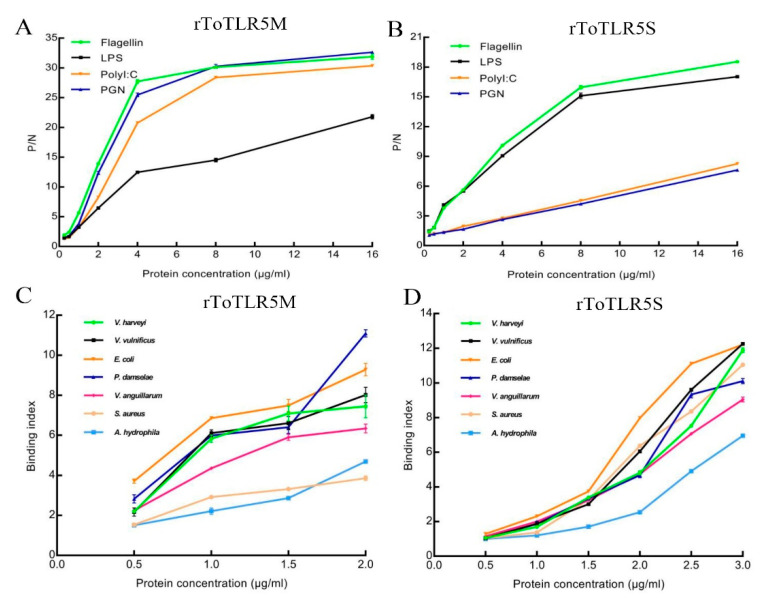
ELISA analysis of the interaction between rToTLR5M (**A**,**C**) and rToTLR5S (**B**,**D**) to pathogen-associated molecular patterns (PAMPs) (**A**,**B**) and bacteria (**C**,**D**), respectively. The microtiter plates were coated with PAMPs and bacteria, and then incubated with different concentrations of recombinant protein. The interaction between protein and PAMPs/bacteria were detected by composite anti-His polyclonal antiserum at 450 nm. Results were representative of an average of three experiments.

The binding of purified two rToTLR5s to bacteria and PAMPs was analyzed by ELISA (Figure 9). The binding activity of two rToTLR5s with Gram-negative bacteria and Gram-positive bacteria was positive, and the binding index was positively correlated with the amount of protein. The binding activity of two ToTLR5 extracellular recombinant proteins to four different PAMPs was positive. rToTLR5M had strong binding activity to flagellin, poly(I:C), and PGN but weak binding activity to LPS (Figure 9A). rToTLR5S had strong binding activity to flagellin and LPS but weak binding activity to poly(I:C) and PGN (Figure 9B). Moreover, rToTLR5M had strong binding activity to *Photobacterium damselae* but weak binding activity to *Aeromonas hydrophila* and *Staphylococcus aureus* (Figure 9C). rToTLR5S had strong binding activity to *Vibrio vulnificus* and *E. coli* but weak binding activity to *Aeromonas hydrophila* (Figure 9D).

## 3. Discussion

The TLR family is highly evolutionarily conserved, and TLRs in most mammals are homologous to those in fish [8]. TLR5 is believed to be the only TLR that binds to the protein PAMP known as bacterial flagellin [11]. The synergistic role of the TLR5 membrane form (TLR5M) and TLR5 soluble form (TLR5S) has been reported in a study on *Onchorhynchus mikiss* [16]. This system is regarded as a unique system in teleost fish. In this study, we cloned both the membrane and soluble form of TLR5 from *T. ovatus*. ToTLR5M, similar to mammalian TLR5, consists of extracellular LRRs, a transmembrane domain, and an intracellular TIR domain. The lack of transmembrane and intracellular TIR domains in ToTLR5S suggests that it can be secreted from cells, as well as other TLR5S proteins in teleost fish [17,18,21,27]. The structural characteristics of these two ToTLR5s were similar to those of the *TLR5* gene in other teleosts [27]. The extracellular LRR domain plays an important role in the recognition of pathogen components on the surface of immune cells. The binding regions of LRR could form on the concave β-face of LRR by a combination of inserts and specific binding surfaces [31,32]. These LRRs seem to be candidates for the flagellin-binding region in two ToTLR5s. The positions of LRR insertions are highly conserved in metazoan (Figure 2A). Therefore, ToTLR5 might be functionally capable of responding to the metazoan TLR5 agonist flagellin. Moreover, in the majority of species, the number of extracellular leucine repeat sequences in TLR is different, which may be attributed to the diversity of species. The intracellular TIR domain of TLR is mainly responsible for signal transmission, including three highly conserved motifs, Box1, Box2 and Box3, in which Box1 and Box2 are associated with signal transduction, whereas Box3 is associated with TLR localization in cells [33].

The tissue expression profile of ToTLR5M mRNA supports previous studies of TLR5M in other fishes, including *I. punctatus*, *Pelteobagrus fulvidraco*, and *O. fasciatus*, which have generally shown similar patterns of constitutive expression in most tissues, with higher expression being observed in the liver [21,34,35]. However, TLR5M had the highest mRNA level in the skin followed by the spleen and kidney in *Epinephelus coioides* [18]. Moreover, TLR5S expression was the highest in the liver followed by the spleen in *E. coioides*, and the transcription of TIL5S was higher in the liver and head kidney than in other tissues in *M. miiuy* [27] and *Scophthalmus maximus* [36], which was different from the results in *T. ovatus*. Liver macrophages are present in the liver, and moreover the kidney and spleen are the main lymphoid organs of fish. These three important tissue sites are involved in the immune response of the body. The expression patterns of the TLR5 gene in various tissues suggested that it plays an important role in the immune monitoring system of these fish, and different tissue expression patterns may be related to species type, individual size, or stage of development.

Previous studies have demonstrated that poly(I:C), LPS, and flagellin can cause defense responses against pathogens in pompano [37,38,39,40,41]. Many studies have found that these PAMPs can induce the expression of *TLR5M* or *TLR5S* genes in immune-related tissues (liver, intestine, blood, kidney, and skin) [17,20,21,36,42,43]. For example, poly(I:C) can increase the mRNA levels of *Carassius auratus TLR5* [42]; LPS can induce the expression of *Pampus argenteus TLR5* [43] and *S. maximus TLR5M* [36]; and flagellin can upregulate the *O. fasciatus*, *P. olivaceus*, and *C. idelus TLR5* expression [17,20,21]. After injections with poly(I:C), LPS, and flagellin, *ToTLR5M* and *ToTLR5S* expression levels were upregulated in the tissues of the immune system, especially in the liver and kidney, which is consistent with *TLR5* expression patterns observed in other fish, such as *O. fasciatus*, *P. olivaceus*, *C. idelus*, *S. maximus*, *C. auratus*, and *P. argenteus* [17,20,21,36,42,43].

The *ToTLR5M* promoter region was located in the -1733 bp to +519 bp region, which included several transcription factor binding sites, such as C/EBP, Sp1, AP-1, Oct-1, NF-кB, c-Rel, and IRF1. The cis-acting elements may be located between −1827 to −1566 bp, −813 to −501 bp, and −309 to +1 bp, which contain NF-кB, GATA-1, C/EBPalpha, NF-1, Oct-1, and ICSBP. This region was similar to that of *O. fasciatus* [21], while the binding sites of AP-1, SP1, SP3, and NF-кB existed in the human *TLR5M* promoter region, and SP1/3 binding sites significantly enhanced TLR5 promoter activity [44]. Moreover, in *ToTLR5S*, the cis-acting elements may be located between −1111 to −802 bp, −490 to −298 bp, and −154 to +1, which contain ICSBP, IRF1, AP-1, GATA-1, C/EBPalpha, and C/EBPbeta. In *Paralichyths olivaceus*, the TLR5S promoter region contained binding sites for such factors as CEBP, AP-1, and NF-кB, and Ap-1 and NF-кB binding sites significantly enhanced TLR5S promoter activity [45]. The transcriptional regulatory mechanisms of TLR5M between *Trachinotus ovatus* and mammals exhibited both similarities and differences, while the TLR5S gene specific to fish had similar activity and was regulated by a variety of transcriptional elements. Future experiments require further analysis of specific transcriptional binding sites.

NF-кB plays an important role in the TLR signaling pathway, and overexpression of *C. idelus* TLR18 and TLR5 has been shown to significantly increase NF-кB activity [42,46]. Furthermore, the TLR5M signaling in response to flagellin abduction in *Onchorhynchus mikiss* is magnified through interaction with the *TLR5S* in a positive loop feedback [8]. In the present study, overexpression of *ToTLR5M* significantly activated NF-кB expression followed by overexpression of *ToTLR5S* or *ToTLR5M* and *ToTLR5S*. It is possible that *ToTLR5M* and *ToTLR5S* have antagonistic effects on the activation of the NF-кB signaling pathway, suggesting that the interactions of *TLR5M* and *TLR5S* were species-specific.

ELISA was used to detect the binding activity of purified protein with bacteria, and it was found that the ToTLR5M and ToTLR5S recombinant proteins containing LRR domains had notable binding activity to Gram-positive/negative bacteria. These results suggest that ToTLR5M and ToTLR5S might recognize and combine pathogenic molecular patterns of some bacteria and play an important role in preventing the infection of Gram-positive/negative bacteria.

The ToTLR5M recombinant protein primarily recognizes the bacterial components peptidoglycan and flagellin and the viral equivalent poly(I:C), while the ToTLR5S recombinant protein primarily recognizes the bacterial components flagellin and liposaccharide. TLR5, located on cell membranes in mammals, primarily recognizes bacterial flagellin [11,47]. Pathogenic bacteria could be identified by TLR5 proteins from fishes such as *E. coioides* [18], *M. miiuy* [27], and *I. punctatus* [34]. The two recombinant proteins have different degrees of binding activity to Gram-negative bacteria and Gram-positive bacteria, indicating that ToTLR5M and ToTLR5S play an important role in antibacterial immune reactions. Moreover, the binding activity of different PAMPs indicated that the extracellular recombinant proteins could participate in the TLR ligand recognition process of pompano. The main ligands recognized by different proteins are different, but the specific binding and mechanism of action warrant further study.

In summary, we identified a membrane form and a soluble form of ToTLR5. Both genes displayed conserved sequence characteristics with those of other fish. The expression analysis of ToTLR5M and ToTLR5S after stimulation with PAMP containing LPS, poly(I:C), and flagellin indicated that the two ToTLR5s played a role in antibacterial immunity. Furthermore, we demonstrated clear associations between NF-кB and the two ToTLR5s promoters, as well as the positive regulatory functions of the two ToTLR5s in NF-кB transcription. ToTLR5M and ToTLR5S were displayed as PRRs that ensured specific binding to various PAMPs and bacteria. The results of the present study indicate that these two ToTLR5s are involved in the immune response to pathogen invasion.

## 4. Materials and Methods

### 4.1. Ethics Statement

In the present study, all trials were approved by the Animal Care and Use Committee of South China Sea Fisheries Research Institute, Chinese Academy of Fishery Sciences (no. SCSFRI96-253, approval date: 11 March 2019) and performed according to the guidelines and regulations established by this committee.

### 4.2. Fish and Challenge Experiments

Juvenile fish (average weight of 40 g) were purchased from Linshui Marine Fish Farm (Hainan, China). The fish were maintained in fresh seawater at approximately 28 °C with 35% salinity and in dissolved oxygen >6 mg/L and were raised 1 week before the trial. Tissue samples (stomach, intestine, kidney, liver, spleen, brain, skin, gill, white muscle, and blood) were collected from six healthy pompano, promptly frozen in liquid nitrogen, and then stored at −80 °C until use.

The stimulation groups were intraperitoneally injected with poly(I:C) (200 µg/mL, 200 µL), flagellin (1 µg/mL, 200 µL), or LPS (50 µg/mL, 200 µL), and the control group was injected with phosphate-buffered saline (PBS, 200 µL). The induction experimental program was described in previous studies [37]. Pompano were anaesthetized using MS222 (0.1 g·L^−1^; Sigma, Alcobendas, Spain) in all groups before tissue sampling. Six tissues (liver, intestine, blood, kidney, skin, and muscle) were harvested from five fish per group at 0 h, 6 h, 12 h, 24 h, 36 h, 48 h, 72 h, and 96 h after the challenge, immediately frozen in liquid nitrogen, and then stored at −80 °C until use.

### 4.3. RNA Extraction and Gene Cloning

Pompano kidney tissues were used to isolate total RNAs (1 µg) by the HiPure Fibrous RNA Plus Kit (Magen, Guangzhou, China). Total RNA was treated with RNase-free DNase I at 37 °C for 30 min and then used to synthesize cDNA by random hexamer primers (Cloned AMV First-Strand cDNA Synthesis Kit, Invitrogen, Carlsbad, CA, USA). The quantity and quality of the extracted RNA were determined by a NanoDrop 2000 spectrometer (Thermo Fisher Scientific, Waltham, MA, USA) and 1% agarose gels. The predicted sequences of *ToTLR5M* and *ToTLR5S* were acquired from pompano genomic data [48]. Moreover, gene-specific primers were designed to amplify the cDNA and genome sequences of two genes that were assembled by SeqMan software of the LaserGene package (DNASTAR, Inc., Madison, WI, USA) (Table 1).

### 4.4. Bioinformatics

Amino acids of two ToTLR5s were used as queries to seek orthologous genes in the NCBI database (http://blast.ncbi.nlm.nih.gov/Blast.cgi). All available TLR5 structures and sequences were provided by Genome Browser (http://genome.ucsc.edu/cgi-bin/hgBlat) and Ensembl (http://asia.ensembl.org/). ClustalW2 (http://www.ebi.ac.uk/Tools/msa/clustalw2/) was used to blast different *TLR5* mature peptide sequences. Compute pI/Mw software (http://web.expasy.org/protparam/) was used to calculate the theoretical isoelectric points and molecular weights.

### 4.5. Cloning of the 5′-flanking Sequence and Its Promoter Activity

Total genomic DNA was extracted from the muscle tissue of pompano according to Sun et al. (2013) [49] and used for cloning of the candidate promoter. To define the core promoter region within the cloned 5′-flanking sequence of *ToTLR5M* and *ToTLR5S*, we amplified seven different promoter regions from *ToTLR5M* and *ToTLR5S* by specific primers with *Hind* III and *Kpn* I restriction sites, respectively (Table 1). Subsequently, the seven truncated fragments of *ToTLR5M* (denoted as ProT5M-1 (−1827 to +565), ProT5M-2 (−1566 to +565), ProT5M-3 (−1131 to +565), ProT5M-4 (−813 to +565), ProT5M-5 (−501 to +565), ProT5M-6 (−309 to +565), and ProT5M-7 (−120 to +565)) were subcloned into the pGL3-basic luciferase reporter plasmid (Promega, WI, USA). Moreover, the seven truncated fragments of *ToTLR5S* (denoted as ProT5S-1 (−1733 to +519), ProT5S-2 (−1358 to +519), ProT5S-3 (−1111 to +519), ProT5S-4 (−802 to +519), ProT5S-5 (−490 to +519), ProT5S-6 (−298 to +519), and ProT5S-7 (−154 to +519)) were also subcloned with the same method. Then, the plasmids of *ToTLR5M* or *ToTLR5S* were transfected into GPS cells.

Furthermore, the ORF of *ToTLR5M* and *ToTLR5S* was cloned into the *Nhe* I and *Kpn* I sites of the pCDNA3.1 vector (Invitrogen, USA). The Renilla luciferase plasmid pRL-TK (Promega, WI, USA) was used as an internal control. The TransGen Plasmid Mini Kit (Beijing, China) was used to isolate recombinant plasmids. GPS culture and transfection experiments were performed according to the methods described by Yu et al. (2016) [50]. Additionally, to further investigate the regulatory relationships between two *ToTLR5s* and ToNF-кB, we also cloned the promoter of *ToNF-кB* into the *Kpn* I and *Xho* I site of the pGL3-basic vector (Invitrogen, USA) (Table 1). Then, ToTLR5M or ToTLR5S or both ToTLR5M and ToTLR5S were transfected into GPS cells together with ToNF-кB.

### 4.6. Quantitative Real-time PCR and Statistical Analysis

The transcription of *ToTLR5M* and *ToTLR5S* was determined by quantitative real-time polymerase chain reaction (qRT-PCR) in 10 healthy tissues and 6 infected tissues in pompano. Total RNA was extracted as described previously. The specific primers of two *ToTLR5s* and the reference gene elongation factor 1 alpha (*EF-1α*) are shown in Table 1. qRT-PCR was performed as previously described [51]. Relative expression was calculated by the 2^−ΔΔCT^ method [52]. SPSS 19.0 software (IBM, NC, USA) was used to analyze the data in the present study. The data were analyzed using the Duncan test by one-way ANOVA from different groups and tissues. Data are presented as the means of three replicates ± standard error (SE), and *p* < 0.05 or *p* < 0.01 indicated significance.

### 4.7. Expression and Purification of Recombinant Two ToTLR5s

The *ToTLR5M* functional domain encoding the LRR region and the ORF of *ToTLR5S* were amplified by PCR with specific primers (Table 1). The corresponding fragments were ligated into the pET-sumo plasmid and transformed into the *E. coli* Rosseta (DE3) strain. Moreover, positive clones were sequenced and confirmed. To abduct the expression of the recombinant proteins of two *ToTLR5s*, we supplied isopropyl-β-D-thiogalactoside (IPTG, 1 mM) at the 0.6 value that the OD600 of primary culture had attained. Bacterial solution (1 mL) was collected for analysis after induction at 37 °C and 220 rpm for 8 h. Furthermore, the control groups were managed analogously without IPTG. According to the His-bind Purification Kit instructions (Novagen), we purified the recombinant proteins of two ToTLR5s and renatured them by Ni-NTA affinity chromatography with chelated nickel ions on the resin. The recombinant proteins were isolated from culture supernatant and separated by sodium dodecyl sulfate polyacrylamide gel electrophoresis (SDS-PAGE; 12% separating gel and 5% stacking gel), observed by staining with Coomassie brilliant blue R250, and detected by Western blotting (WT) analysis. A Modified BCA Protein Assay Kit (Sangon Biotech, Shanghai, China) was used to measure the concentration of the ToTLR5M and ToTLR5S proteins.

WT analysis was performed after the purified recombinant ToTLR5M (rToTLR5M) and ToTLR5S (rToTLR5S) was separated by 12% SDS-PAGE. The outcomes were transferred onto nitrocellulose membrane, washed with PBST (phosphate buffer solution Tween; 0.1 M) thrice, and blocked with a suspension of 5% skim milk powder for 2 h. Subsequently, PBST was used to wash the membrane and it was then incubated with His-Tag horseradish peroxidase (HRP) (1:2000) conjugated mouse monoclonal antibody at 37 °C for 1 h. Enhanced HRP-DAB substrate color development kit was used to develop the immune reactive bands.

### 4.8. Assay for the Binding of Bacteria and PAMPs

To investigate the binding ability of recombinant ToTLR5M (rToTLR5M) and ToTLR5S (rToTLR5S) protein with bacteria and PAMPs, we determined six types of bacteria (*Vibrio harveyi*, *Vibrio vulnificus*, *Vibrio anguillarum*, *Staphylococcus aureus*, *Escherichia coli*, *Aeromonas hydrophila*, *Photobacterium damselae*) and four types of PAMPs (poly(I:C), LPS, flagellin, and peptidoglycan (PGN); Sigma-Aldrich, USA) by enzyme-linked immunosorbent assay (ELISA). The bacteria were harvested by centrifugation at 12,000× *g* for 5 min after cultivation, washed with PBS three times, and suspended to approximately 1 × 10^8^ cfu/mL with PBS. Bacterium and PAMPs were added to each well of a microtiter plate and maintained at 4 °C for 24 h. The plates were washed three times and then 100 μL of various concentrations of recombinant protein or BSA (as a negative control) was added to the wells. Monoclonal His-Tag antibody (1:1000 dilution) was added to each well and incubated at 37 °C for 1 h. The detailed procedure was described as reported previously [53]. The binding activity of rToTLR5M and rToTLR5S to bacteria or PAMPs was calculated by the OD450 ratio of the treatment and control groups. Positive readings were defined as at least twice that of the control. All assays were implemented in triplicate.

## Figures and Tables

**Table 1:** Primers used for sequence cloning, deletion mutant construction, mRNA construction, and qRT-PCR.

Subject and Primers	Nucleotide Sequence
**Primers for cDNA sequence cloning**	
TLR5M-F	GCTAGCATGAGGACGCCGGCCCTTCACT
TLR5M-R	GGTACCTCACATAGCAATTGTTGGGATG
TLR5S-F	GCTAGCATGTGGCTGCTGGGTCTCCAGG
TLR5S-R	GGTACCTTACTGCTGTGTGAGCTGAGCA
TLR5M-GSP1	GACACCAAGGTGAAAAAGATTG
TLR5M-GSP2	GACCAAAAGTAGTGTATTCATA
TLR5S-GSP1	ACCAATGTCACCTTCTTGAGTC
TLR5S-GSP2	ACGTATGTCATGTTGATTTGGG
**Primers for DNA sequence cloning**	
TLR5M-F1	CAACAGCAAAGTTAGATTTACCAAT
TLR5M-R1	CAATTTGAGGTTTAATTGTGTGAAC
TLR5M-F2	AGAGACTCCATACTGACGGAAAGC
TLR5M-R2	AGGAACATGCTATATATCAGCTGT
TLR5M-F3	AAATATGAAGCTACAGCCAGACGC
TLR5M-R3	TGTGAGATTTTGTTTTAATTCTTA
TLR5S-F1	GTTTGTGCTGCACAATCACAGTAATG
TLR5S-R1	CCGTTGTAGGATAGGTCGAGTTTCTG
TLR5S-F2	TCAATTTCCTAAACTTGAACTCTG
TLR5S-R2	GCCCATTTCAGGGGATTTTTTTAT
**Deletion mutant construction**	
Pro-TLR5M-F1	AAGCTTACCTGCGGTATGGAAGAATGCCCTG
Pro-TLR5M-F2	AAGCTTACCCTCGCACATCACTTCCTTAACC
Pro-TLR5M-F3	AAGCTTACCTAGCTACGGAGGACAGGACTG
Pro-TLR5M-F4	AAGCTTACCAGGTCGGCCGCCCAGAAACC
Pro-TLR5M-F5	AAGCTTACCGACTATGGAAAAGTTACAAG
Pro-TLR5M-F6	AAGCTTACCTACAAGTTAAAGAGCAGAGAG
Pro-TLR5M-F7	AAGCTTACCGAAATGCTCCAGGCGGGTCA
Pro-TLR5M-R	GGTACCTTAGCTGTCCTCGTCCCACCAAGGCG
Pro-TLR5S-F1	AAGCTTACCGGTCTTTCACTGACTTCCCTAC
Pro-TLR5S-F2	AAGCTTACCTCCAGTGGTGAAAAAGCAGCTG
Pro-TLR5S-F3	AAGCTTACCACCACTCTCTTTAATTATTTACAG
Pro-TLR5S-F4	AAGCTTACCCATTGGGCGCTCAGAAATCACTTG
Pro-TLR5S-F5	AAGCTTACCCTCCTACTCCTAAAAGTACAATA
Pro-TLR5S-F6	AAGCTTACCGTCTTAATATCTGAAAGAGGAA
Pro-TLR5S-F7	AAGCTTACCGATGCTGTTTTCTGTACTACTGAC
Pro-TLR5S-R	GGTACCTTACCTCACACTGCTTGGTATAATCC
Pro-NF-кB-F	GGTACCGTAAGATCATGTGAACTACC
Pro-NF-кB-R	CTCGAGGTATGAAGGTAGTGGTCGTC
**Primers for qRT-PCR**	
qTLR5M-F	TTCAGTCACTCATCTTCCTCAG
qTLR5M -R	TCTCGTTCAGCCACTTCAG
qTLR5S-F	TCAACCTCTCCAACAACTTCA
qTLR5S-R	CGGTCATCCAAGCCAGAA
EF1α-F	AAGCCAGGTATGGTTGTCAACTTT
EF1α-R	CGTGGTGCATCTCCACAGACT
**Primers for recombinant expression**	
TLR5M-ED-F	GGATCCGAATTCCGGACTATGCTGGAGCCAAGGGCATTTGC
TLR5M-ED-R	GTGGTGCTCGAGTGCGGCCTTATTAATCCTCTTTACATGGTTCGATG
TLR5S-ED-F	GGATCCGAATTCCGGACTATGTCATGCCTCATAACGGGCTC
TLR5S-ED-R	GTGGTGCTCGAGTGCGGCCTTATTACTGCTGTGTGAGCTGAGCAG
**Primers for eukaryotic recombinant vector construction**	
TLR5M-F	TGGACTAGTGGATCCATGAGGACGCCGGCCCTT
TLR5M-R	TTTAAACTTAAGCTTCATAGCAATTGTTGGGAT
TLR5S-F	TGGACTAGTGGATCCATGTGGCTGCTGGGTCTC
TLR5S-R	TTTAAACTTAAGCTTCTGCTGTGTGAGCTGAGC

The underline indicates restriction enzyme cutting sites.

## Supplementary Materials

Supplementary materials can be found at https://www.mdpi.com/1422-0067/21/16/5916/s1.

## Abbreviations

TLRs	Toll-like receptors
ORF	open reading frame
poly(I:C)	polyinosinic:polycytidylic acid
LPS	lipopolysaccharide
PAMPs	pathogen-associated molecular patterns
PGN	peptidoglycan
PRR	pathogen recognition receptor
LRRs	leucine-rich repeats
GPS	*Trachinotus ovatus* snout tissue
qRT-PCR	quantitative real-time polymerase chain reaction
WT	Western blotting
SDS-PAGE	sodium dodecyl sulfate polyacrylamide gel electrophoresis
ELISA	enzyme-linked immunosorbent assay
