# Supplementary material for: Toll-Like Receptor 5 of Golden Pompano Trachinotus ovatus (Linnaeus 1758): Characterization, Promoter Activity and Functional Analysis
Source: Int J Mol Sci. 2020 Aug 18;21(16):5916. doi: 10.3390/ijms21165916 (PMC7460618; doi:10.3390/ijms21165916)
Supplement: Supplementary file 1 [file ijms-21-05916-s001.pdf]

Supplementary Materials:

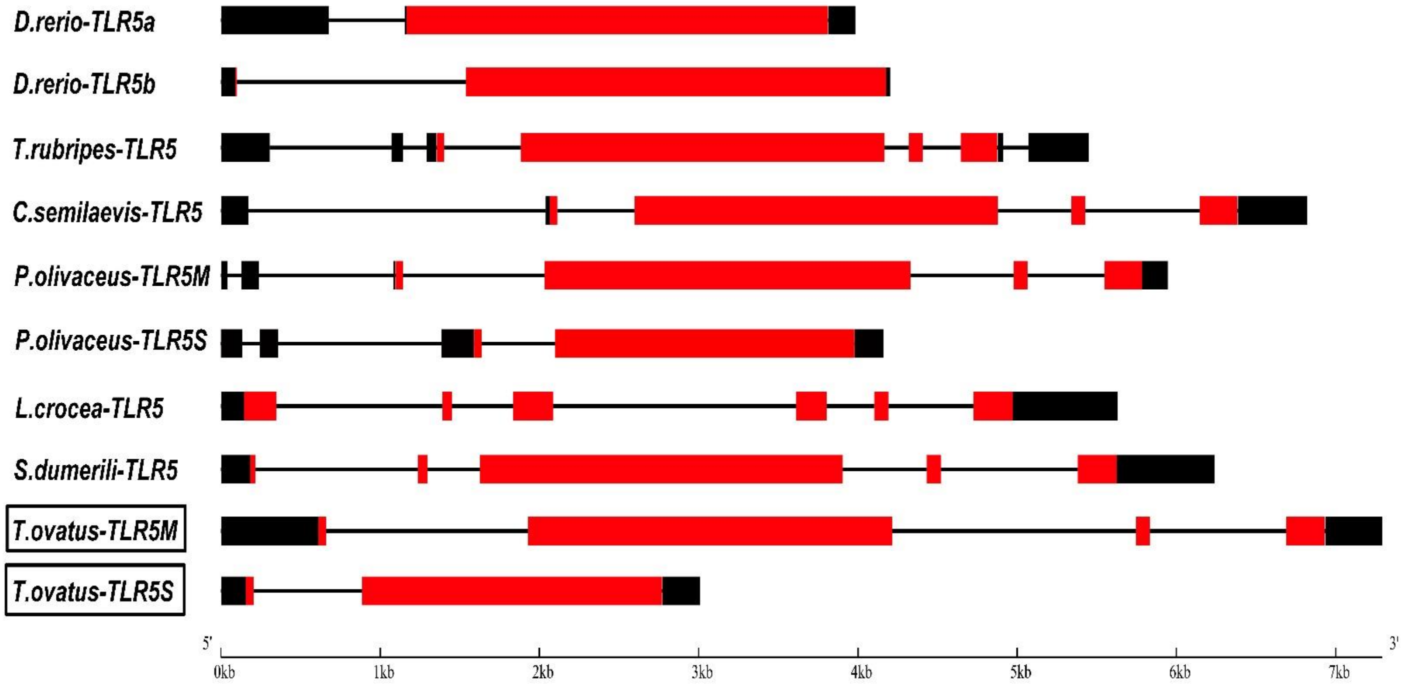

Figure S1. Comparison of the exon-intron organization of the *TLR5*. The boxes and bars represented the exons and introns, respectively. The boxes in black represented the UTR. The boxes in red represented the CDS. The TLR5 sequence No. of each species: *D. rerio* (NC_007131.7); *T. rubripes* (NC_018905.1); *C. semilaevis* (NW_007585113.1); *P. olivaceus* (TLR5M：NW_017859659.1; TLR5S：NW_017860348.1); *L. crocea* (NC_040021.1); *S. dumerili* (NW_019174280.1).

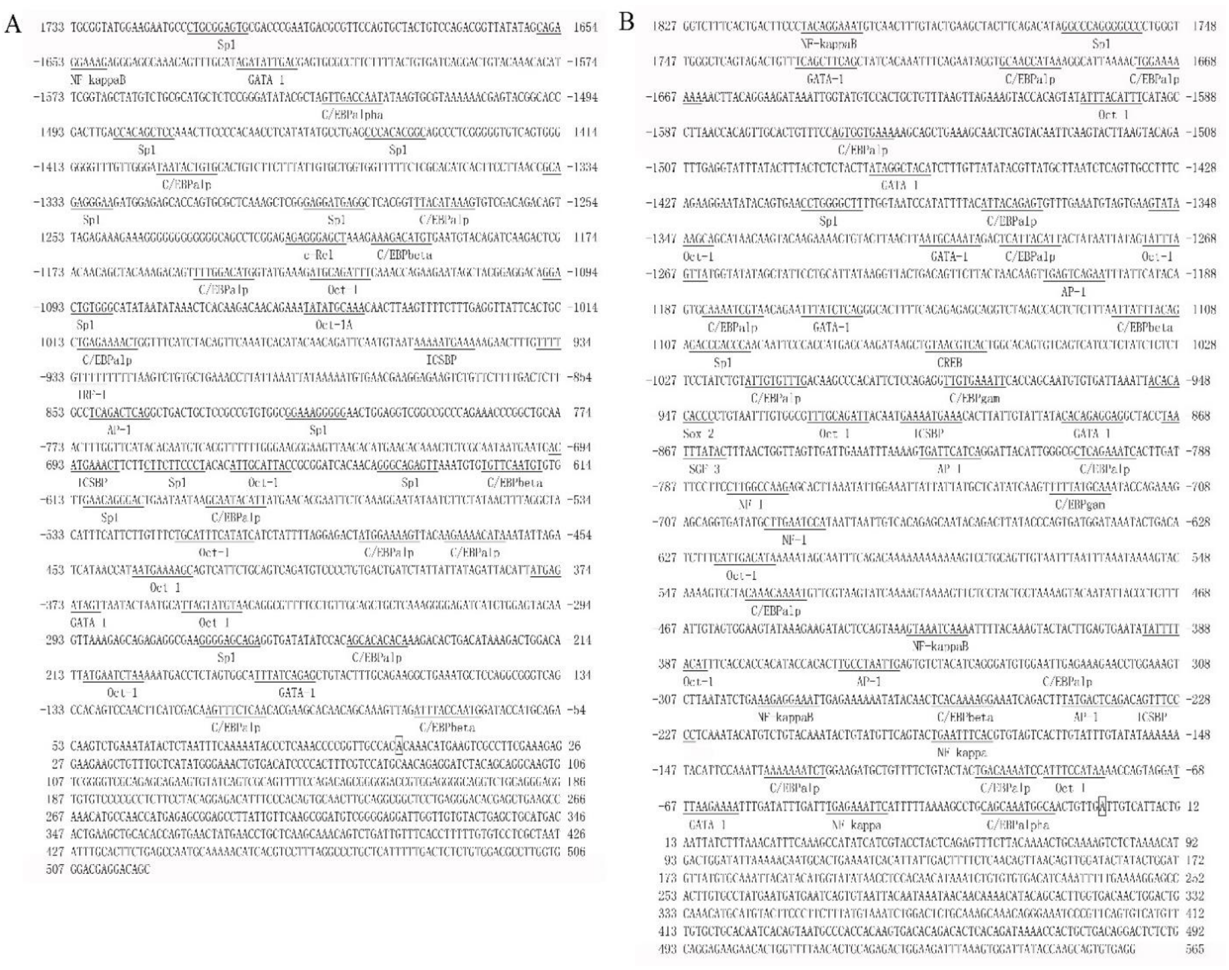

Figure S2. Analysis of the promoter sequences of *ToTLR5M* (A) and *ToTLR5S* (B). Nucleotide sequence of 5′-flanking regions of two *ToTLR5s*. The predicted transcriptional start site was expressed in a box and defined as +1; the predicted transcription factor binding sites were underlined. The box showed the transcriptional start site.

Table S1 The amino acid sequences in various species used for domain structures.

TLR	Species	Accession Number
TLR5	*Homo sapiens*	AAI09119.1
	Mus musculus	AAI25248.1
	Gallus gallus	ACR26275.1
	Danio rerio	AAI63198.1
	Cyprinus carpio	BAU98383.1
	Takifugu rubripes	AAW69374.1
TLR5M	*Trachinotus ovatus*	MT596697
	Coregonus maraena	CEF90216.1
	Epinephelus coioides	AIS23536.1
	Miichthys miiuy	ALJ55566.1
	Paralichthys olivaceus	BAJ16367.1
TLR5S	*Trachinotus ovatus*	MT596698
	Ictalurus punctatus	AEI59667.1
	Epinephelus coioides	ACV04459.1
	Miichthys miiuy	ALJ55567.1
	Paralichthys olivaceus	BAJ16368.1
